# Personality over ontogeny in zebra finches: long-term repeatable traits but unstable behavioural syndromes

**DOI:** 10.1186/1742-9994-12-S1-S9

**Published:** 2015-08-24

**Authors:** Yvonne Wuerz, Oliver Krüger

**Affiliations:** 1 Department of Animal Behaviour, Bielefeld University, PO Box 100131, 33501 Bielefeld Germany

**Keywords:** Animal personality, behavioural syndrome, repeatability, consistency, ontogeny, tonic immobility, exploration, boldness, aggression, activity

## Abstract

A crucial assumption of animal personality research is that behaviour is consistent over time, showing a high repeatability within individuals. This assumption is often made, sometimes tested using short time intervals between behavioural tests, but rarely thoroughly investigated across long time intervals crossing different stages of ontogeny. We performed such a longitudinal test across three life stages in zebra finches (*Taeniopygia guttata*), representing about 15-20% of their life span in captivity, and found repeatabilities ranging from 0.03 to 0.67. Fearlessness and exploration were the most repeatable traits both within and across life stages. Activity and aggression were repeatable across, but not or only partly within life stages. Boldness was not repeatable. Furthermore, we found no evidence for a consistent behavioural syndrome structure across ontogeny. Our results indicate that the consistency of behavioural traits and their correlations might be overestimated and suggest that life-long stability of animal personality should not simply be assumed.

## Background

Animal personality by definition focuses on stable between-individual differences in behaviour, being 'consistent over time and/or contexts' [1,2]. It has been shown to influence fitness [3] and occurs in a wide array of vertebrate and invertebrate taxa [4,5]. Consistency can apply to two aspects of personality, with single traits being repeatable ('differential consistency') and multiple traits being correlated ('structural consistency') [6].

### Repeatability ('differential consistency')

If a single trait is stable over time, it has a high repeatability. Repeatability is the proportion of variance in a given trait that can be attributed to the difference between individuals (and is thus always a population level measure [7]). That is, when multiple measurements of the same individual are similar (low within-individual variance), while at the same time individuals differ substantially from each other (high between-individual variance), the behavioural variable is repeatable.

Average repeatability of behavioural traits has been shown to be around 0.4 [8] and 0.48 [9] in two meta-analyses. It can be influenced by several factors, for instance the type of behaviour, the time interval between tests, age or ontogenetic stage and the sex of the individuals [8]. Unfortunately, the vast majority of studies merely use two measurements per individual often quantified over short time scales. This was indicated in a meta-analysis [8], with a mode of 2 and a mean of 4.4 measures per individual over 759 studies [10] and only 9% of these studies covering a 'long' time interval, defined as over one year. This seems rather insufficient, as especially for traits with low repeatability, we expect the precision of the estimate to increase with higher sample sizes (number of subjects and/or test rounds) [9].

### Behavioural syndrome structure ('structural consistency')

Not only can single traits show consistency, but also the between-individual correlations of two or more behavioural traits, forming a behavioural syndrome. Behavioural syndromes represent a functional coupling of traits [11] which has been argued to be either adaptive [12-14] or maladaptive [1,15,16]. They can emerge quickly and may change over time, or with critical life experiences [17-19], though a meta-analysis on behavioural syndromes found on average weak phenotypic correlations between behavioural traits (mean effect size r = 0.264 with 95% CI 0.210-0.316) [20].

### Investigating personality over ontogeny

Although consistency is widely acknowledged as an important prerequisite for animal personality, studies often identify and describe animal personality and behavioural syndromes at a single point in time (and possibly their implications e.g. on fitness, or correlations to physiological parameters [21]), leading to a gap in our understanding how animal personality develops. Even when personality is tested over time, the test-retest intervals are mostly short [8] and not representative of the species' life span and different life history stages [6]. So far, most studies only dealt with adult animals, providing an incomplete picture of animal personality [but see e.g. [22-25]] and resulting in misinterpretations of the consistency of personality. This lack of studies across key stages of ontogeny probably arises from the intuitive contradiction between the two topics, with personality focusing on stability, while ontogeny deals with how organisms change over time [26]. However, there have been recent calls for a broader incorporation of developmental aspects (measurements over different ontogenetic stages) into animal personality research [27-29], arguing that this could reveal the proximate mechanisms underlying personalities. As recent findings indicate mixed evidence with regard to the consistency of personality over ontogeny ((partly) inconsistent: [15,17,30], consistent: [23,31]), this challenges the assumption that personality is fixed across a lifetime.

Even though the literature focusing on ontogenetic stages currently increases significantly, few studies assess more than two (or three) personality traits (but see e.g. [32]). Furthermore, only little is known about how behavioural syndrome structure changes over time. Juveniles are facing different challenges (ecological or social niches) compared with adults (e.g. their behaviour should focus on survival rather than reproduction), hence adaptations needed for early life stages might disappear later [29], resulting in behavioural syndromes either forming or decoupling during adolescence. If the behaviours forming a syndrome are regulated by a common underlying physiological mechanism, they might be linked particularly tightly, potentially imposing a developmental constraint and thus limiting optimal behaviour, because single traits cannot change independently [15]. Indeed, behavioural syndrome structure was shown to be consistent over time (two weeks) [32] or similar across two cohorts [33].

So far, the existence of animal personality and behavioural syndromes in general is well documented, leading to a current shift to investigate the causes and consequences of personality [34,35]. One of the most extensively studied model organisms is the zebra finch, *Taeniopygia guttata*. In this species, several personality traits have been identified, such as activity [22,36,37], aggression [37,38], fearfulness [36], exploratory behaviour and struggling rate [37,39] and boldness/neophobia [37,40] including their heritability [40]. Furthermore, the existence of behavioural syndromes has been documented in zebra finches [37], as well as implications of personality type for fitness correlates e.g. reproductive success [41,42].

### Aims of this study

With our study, we intended to address the following widespread shortcomings regarding consistency of animal personality: (a) testing over short intervals or within single developmental stages, neglecting (b) the aspect of multidimensionality (assessing only one or two traits) and (c) the development of behavioural syndrome structure.

Our aim was to assess long-term behavioural consistency in zebra finches (*Taeniopygia guttata*) over ontogeny. The birds chosen for this study originated from selection line experiments on animal personality. We investigated both differential consistency (repeatability) and structural consistency (behavioural syndrome structure) in five different behavioural tests for three life stages in both sexes. We conducted tests on fearlessness (tonic immobility), aggression against a mirror, general activity in the home cage, boldness towards a novel object and exploration of a novel environment. Testing began shortly after independence from the parents ('subadult' stage, about 55 days old), and was repeated at 'young adult' (100 days old) and 'mature adult' (1 year old) stages. Two test rounds were conducted within each life stage, resulting in six test rounds in total (see Table 1 for age at test rounds and intervals between rounds). Reproductive maturation commences at the stage that we referred to as 'subadult'. Zann [43] reported the median age of birds breeding within the same season they hatched as 95 d for males and 92 d for females, which in our experiments is equivalent to test round 3 in the 'young adult' stage. The 'young adult' stage hence represents the (potential) beginning of reproductive activity.

**Table 1:** Description of test rounds with mean age at the beginning of tests and interval from the preceding test round.

Life stage	Test round	Age [mean ± SD]	Interval [days]
Subadult	1	56 ± 7	-
	
	2	73 ± 8	17 ± 7

Young adult	3	103 ± 5	31 ± 6
	
	4	121 ± 5	18 ± 4

Mature adult	5	367 ± 52	247 ± 52
	
	6	381 ± 51	14 ± 0

Maximum life spans of 1.3 to 5 years have been reported for different zebra finch populations in the wild [43], while under laboratory conditions, life spans may be at least 5 to 9 years [44,45].

We repeated our tests after approximately one year ('mature adult' stage), which is a long time span in zebra finches, representing about 15 - 20% of the lifespan of captive animals. So far, the longest test-retest intervals in zebra finches we are aware of is three to seven months for exploration behaviour [39,42], seven months for struggling rate [39], 1.5 years for neophilia [40] and even two years for activity levels [22].

We therefore provide the first extensive longitudinal study on a multitude of personality tests, following the same individuals over a substantial amount of their lifespan. We specifically asked the following questions: (1) How stable are the single personality traits within and across key ontogenetic stages; (2) which factors influence repeatability (trait, interval, life stage, sex); (3) how stable are the correlations between the traits (behavioural syndromes)?

## Results

For descriptive statistics of the untransformed behavioural variables measured, see part 1 of additional file 1 and for the results and loadings of the principal component analyses, see part 2 of additional file 1.

### Repeatabilities ('differential consistency')

**Figure 1:**
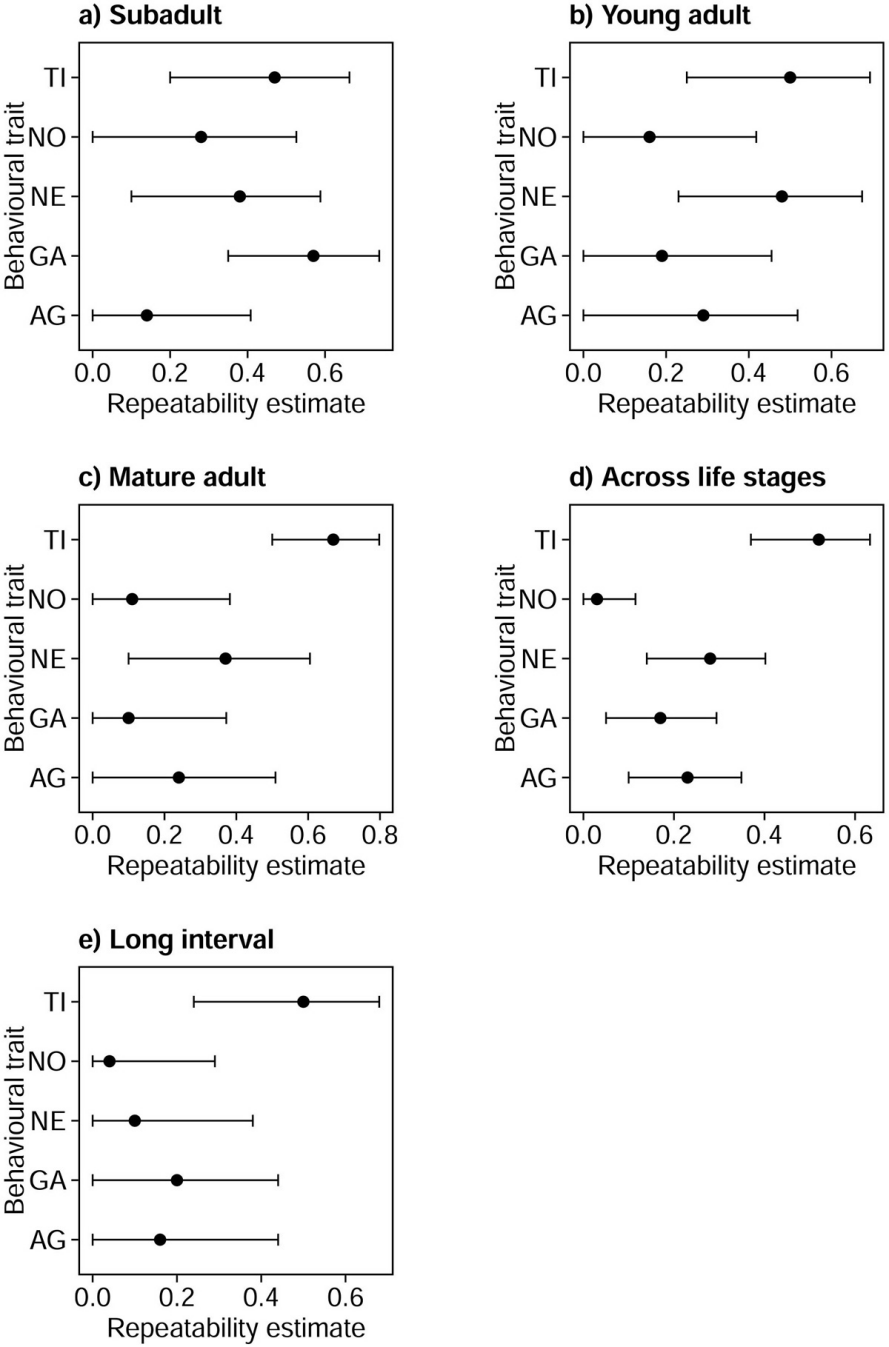
****Repeatabilities of PC axes, with their 95% confidence intervals, within and across the three life stages**** for the five behavioural traits (TI = Fearlessness, NO = Boldness, NE = Exploration, GA = General activity, AG = Aggression) in (a) subadults, (b) young adults, (c) mature adults (d) across life stages including all six measurements per individual and (e) across a long interval including only the measurements from test rounds 1 and 6. Overlapping confidence intervals indicate non-significant differences between repeatability estimates.

Repeatability estimates are presented in Figure 1. Fearlessness (tonic immobility test, TI) and exploration (novel environment test, NE) were repeatable within all life stages and across the whole measurement period including all six test rounds. TI, however, was the only trait with a significant repeatability if the estimate was derived from only two data points with an interval of about one year ('long interval' using test rounds 1 and 6).

General activity (GA) was repeatable within the subadult stage and across all life stages, but not in young or mature adults.

In general, repeatability estimates for boldness (novel object test, NO) were very low (R ranging between 0.03 and 0.28), which might be due to the differences in the novel objects used in the test rounds (for details see table 1). NO in subadults and aggression (AG) in young adults showed significant p-values but confidence intervals overlapping with zero indicated non-significance. AG repeatability estimates were significant across life stages, but not within life stages.

### Sex differences in repeatability

For some traits at some life stages, we found that repeatability differed between the sexes (for further details see table 2). For TI, repeatability estimates were highly significant at all life stages, and the estimates did not differ between males and females. NE was not repeatable in males at the subadult and young adult life stages, but it was in mature adults. Conversely, females had repeatable NE estimates at subadult and young adult life stages but not at the mature adult stage. The full dataset across all life stages revealed a significant repeatability only for females, but not males.

**Table 2:** Repeatabilities of PC axes, with their 95% confidence intervals, within and across the three life stages, calculated for all individuals and separately for the sexes.

		Subadult (1-2)			Young adult (3-4)			Mature adult (5-6)			Across life stages (1-6)			Long interval (1 vs 6)		
**Personality trait**	**Sex**	***R***	**CI (95%)**	***p***	***R***	**CI (95%)**	***p***	***R***	**CI (95%)**	***p***	***R***	**CI (95%)**	***p***	***R***	**CI (95%)**	***p***

Fearlessness																

	both	**0.47**	0.20-0.66	0.001	**0.50**	0.25-0.69	0.001	**0.67**	0.50-0.80	0.001	**0.52**	0.37-0.63	0.001	**0.50**	0.24-0.68	0.001

	males	**0.42**	0.03-0.71	0.022	**0.46**	0.08-0.74	0.008	**0.68**	0.32-0.85	0.001	**0.52**	0.31-0.67	0.001	**0.55**	0.19-0.78	0.005

	females	**0.51**	0.17-0.74	0.01	**0.58**	0.23-0.79	0.003	**0.68**	0.40-0.84	0.001	**0.52**	0.31-0.67	0.001	**0.47**	0.10-0.73	0.009

Exploration																

	both	**0.38**	0.10-0.59	0.013	**0.48**	0.23-0.67	0.001	**0.37**	0.10-0.61	0.005	**0.28**	0.14-0.40	0.001	0.10	0-0.38	> 0.05

	males	0.21	0-0.58	> 0.05	0.23	0-0.59	> 0.05	**0.43**	0.004-0.70	0.026	0.05	0-0.19	>0.05	0.00	0-0.44	> 0.05

	females	**0.41**	0.04-0.69	0.028	**0.60**	0.29-0.79	0.002	0.25	0-0.58	>0.05	**0.36**	0.15-0.52	0.001	0.21	0-0.54	> 0.05

Boldness																

	both	**0.28**	0-0.53	0.018	0.16	0-0.42	> 0.05	0.11	0-0.38	> 0.05	0.03	0-0.12	> 0.05	0.04	0-0.29	> 0.05

	males	0.25	0-0.61	> 0.05	0.12	0-0.53	>0.05	0.24	0-0.60	>0.05	0.09	0-0.25	> 0.05	0.00	0-0.39	> 0.05

	females	0.30	0-0.61	> 0.05	0.20	0-0.55	>0.05	0.00	0-0.40	>0.05	0.00	0-0.11	>0.05	0.12	0-0.48	> 0.05

Activity																

	both	**0.57**	0.35-0.74	0.001	0.19	0-0.46	> 0.05	0.10	0-0.37	> 0.05	**0.17**	0.05-0.29	0.001	0.20	0-0.44	0.08

	males	**0.70**	0.39-0.86	0.002	0.00	0-0.40	>0.05	0.04	0-0.45	> 0.05	0.12	0-0.27	0.042	0.19	0-0.57	> 0.05

	females	**0.48**	0.13-0.72	0.011	0.34	0-0.64	0.046	0.13	0-0.51	> 0.05	**0.20**	0.04-0.36	0.002	0.22	0-0.55	> 0.05

Aggression																

	both	0.14	0-0.41	> 0.05	0.29	0-0.52	0.021	0.24	0-0.51	> 0.05	**0.23**	0.10-0.35	0.001	0.16	0-0.44	> 0.05

	males	0.00	0-0.04	> 0.05	0.31	0-0.66	> 0.05	0.10	0-0.48	> 0.05	0.10	0-0.26	0.03	0.05	0-0.45	> 0.05

	females	0.34	0-0.64	0.044	0.29	0-0.58	> 0.05	0.36	0-0.67	0.038	**0.33**	0.14-0.50	0.001	0.26	0-0.59	> 0.05

Significant repeatability estimates are in bold.

GA was highly repeatable for both males and females at the subadult life stage, but at the young adult stage only for females, and in mature adults in neither of the sexes. Aggression (AG) repeatability estimates of males and females showed confidence intervals overlapping with zero within all life stages. Across life stages, only female aggression was repeatable. NO repeatability estimates were similar for the sexes and non-significant in all life stages.

### Behavioural syndrome structure ('structural consistency')

No significant correlations between fearlessness, aggression, activity, boldness and/or exploration were found at the subadult and mature life stages. Only the young adult life stage revealed a behavioural syndrome consisting of aggression, activity and shyness. While activity and aggression were positively correlated (r = 0.49, CI 0.23 - 0.68, p < 0.001), both activity and boldness (r = -0.56, CI -0.73 - (-0.32), p < 0.001) and boldness and aggression (r = -0.57, CI -0.74 - (-0.34), p < 0.001) were negatively correlated. Thus, more active individuals were also more aggressive and less willing to approach a novel object. However, the syndrome structure changed between life stage transitions, so that none of the correlations was stable over the whole measurement period. A detailed correlation matrix is given in table 3.

**Table 3:** Behavioural syndrome structure at three life stages, with correlation coefficients below diagonal and 95% confidence intervals above diagonal.

Subadult					
	**TI**	**AG**	**GA**	**NO**	**NE**

Fearlessness (TI)		-0.24 - 0.34	-0.22 - 0.36	-0.30 - 0.28	-0.31 - 0.27

Aggression (AG)	0.05		-0.25 - 0.33	-0.19 - 0.39	-0.35 - 0.22

General activity (GA)	0.08	0.04		0.07 - 0.58	-0.13 - 0.44

Boldness (NO)	-0.01	0.11	0.35		-0.03 - 0.51

Exploration (NE)	-0.02	-0.07	0.17	0.26	

**Young adult**					

	**TI**	**AG**	**GA**	**NO**	**NE**

Fearlessness (TI)		-0.28 - 0.30	-0.57 - (-0.06)	-0.09 - 0.47	-0.27 - 0.31

Aggression (AG)	0.01		**0.23 - 0.68**	**-0.74 - (-0.34)**	-0.22 - 0.36

General activity (GA)	-0.34	0.49**		**-0.73 - (-0.32)**	-0.34 - 0.24

Boldness (NO)	0.21	-0.57**	-0.56**		0.01 - 0.54

Exploration (NE)	0.02	0.08	-0.05	0.30	

**Mature adult**					

	**TI**	**AG**	**GA**	**NO**	**NE**

Fearlessness (TI)		-0.39 - 0.19	-0.39 - 0.19	-0.28 - 0.30	-0.26 - 0.32

Aggression (AG)	-0.11		-0.46 - 0.10	-0.06 - 0.49	-0.08 - 0.48

General activity (GA)	-0.11	-0.20		-0.16 - 0.41	-0.01 - 0.53

Boldness (NO)	0.01	0.23	0.14		-0.13 - 0.44

Exploration (NE)	0.03	0.22	0.28	0.17	

Spearman rank correlations were calculated for each life stage with the mean of fitted values from the models (BLUPs). P-values were adjusted for multiple testing with Holm's correction. Significant correlations are in bold. *p < 0.05; ** p < 0.01

We additionally calculated behavioural syndrome structures separately for the sexes. Comparison with the dataset for both sexes revealed that the significant correlations in young adults seem to be highly driven by the female sex. While the correlations for females were similar, but with higher correlation coefficients (AG-NO: r = -0.69; CI -0.85 - (-0.41); p < 0.001; GA-NO: r = -0.61; CI -0.81 - (-0.28); p < 0.01 and GA-AG: r = 0.53; CI 0.17 - 0.76; p < 0.05, for more details see part 3 in additional file 1), these correlations were completely absent in the male subset. There were no significant correlations for males, except for a positive trend between exploration and aggression (r = 0.57; CI 0.18 - 0.80; p = 0.07) at the mature life stage.

### Generalized linear mixed effects models

Results of the GLMMs are given in detail in part 4 (complete, 'inflated' datasets caused by zero- and maximum-inflated data) and part 5 ('reduced' datasets excluding these floor or ceiling effects) of additional file 1. Only in a few cases did we find evidence for significant effects of test round and sex. Fixed effects are given in brackets as estimates ± SE.

For fearlessness (TI), the interaction between sex and test round was (marginally) significant at the mature life stage in the inflated dataset (0.61 ± 0.30, t_13 _= 2.01, p = 0.05), but not the reduced dataset (0.07 ± 0.21, t_11 _= 0.33, p = 0.75). Conversely, at the young adult life stage, the interaction was significant in the reduced dataset (0.44 ± 0.21, t_13 _= 2.15, p = 0.03), but not in the inflated dataset (0.48 ± 0.34, t_11 _= 1.39, p = 0.17).

In young adults, aggression (AG) seemed to be lower in round 4 (-0.22 ± 0.11, t_11 _= -2.05, p = 0.04), but this effect disappeared in the reduced dataset (-0.13 ± 0.10, t_11 _= -1.27, p = 0.21).

Young adult males were more active than females (GA reduced dataset: 0.36 ± 0.15, t_12 _= 2.36, p = 0.02; inflated dataset: 0.51 ± 0.27, t_11 _= 1.84, p = 0.07).

Boldness (NO) was influenced by round, but this effect only became apparent in the reduced datasets, but not in the zero-inflated dataset. Thus, after removal of the birds that did not approach the novel object at all from the analysis, subadults were less bold in round 2 where a white object was used (estimate from unweighted model: -0.96 ± 0.21, t_11 _= -4.54, p < 0.001). At both the young and mature adult stages, there was an increase in boldness between test rounds, when blue objects were used (estimates from unweighted models; young adult: 2.12 ± 0.26, t_12 _= 8.16, p < 0.001; mature adult: 0.91 ± 0.41, t_12 _= 2.24, p = 0.03). Furthermore, males were bolder than females at the young and mature adult life stages (young adult: 0.55 ± 0.24, t_12 _= 2.29, p = 0.03; mature adult: 1.06 ± 0.39, t_12 _= 2.72, p = 0.01). At the subadult stage, sex was only marginally significant (0.41 ± 0.22, t_11 _= 1.85, p = 0.07). All these effects were not detected in the inflated datasets.

Exploration behaviour (NE) was influenced by sex at the mature life stage, with males being more explorative than females. This effect was significant both for the inflated dataset (0.84 ± 0.31, t_12 _= 2.69, p = 0.01) and reduced dataset (0.49 ± 0.11, t_12 _= 4.29, p < 0.001).

### Effect of selection line origin

The birds chosen for this study originated from selection line experiments on animal personality. However, as they were from the F1-generation, we did not expect pronounced differences between lines yet, which is supported by the fact that line was not significant in most models except for NO at the subadult stage. Birds from the lines selected on low NE and high TI were less bold (estimates from unweighted model: NE low: -1.10 ± 0.52, t_11 _= -2.13; TI high: -1.47 ± 0.66, t_11 _= -2.22; p = 0.03). This effect of line did not occur in the GLMM using the reduced dataset without floor and ceiling effects.

## Discussion

### Repeatabilities ('differential consistency')

We found evidence for considerable differences in repeatability of the five behavioural traits within and across life stages. We showed that in zebra finches, fearlessness (TI) and exploration (NE) were highly repeatable both within and across life stages. General activity (GA) and aggression (AG) were only repeatable at specific life stages and also across life stages, while boldness (NO) was not repeatable at all. Thus our results are within the range of repeatabilities described for behavioural traits in general [8,9].

The factors possibly influencing repeatability include the type of behaviour, the interval between observations, age and sex of the individuals, which we will discuss in the following.

### Behavioural traits differ in their repeatability

A categorization of behaviours into functional classes showed that exploratory behaviour and aggression were among the most repeatable traits, while activity was one of the least repeatable [8]. Our results partly confirm this as exploratory behaviour (NE) was highly repeatable throughout, and activity (GA) was only repeatable in subadults. In contrast to previous findings, we found aggression to be of only low repeatability across life stages, and non-repeatable within life stages. This might be explained by the fact that zebra finches are a highly gregarious species with comparatively low levels of aggression, hence there might be little need for consistent aggressive behaviour e.g. to assess or stabilize a dominance hierarchy through aggressive confrontations.

Tonic immobility (TI) has been described as the endpoint of a reflexive defence cascade in response to predators occurring in a wide range of animals and also in humans [46]. It represents an unlearned response reducing the likelihood of being attacked or increasing the survival after a predator attack [47] which seems to be evolutionarily conserved. Because TI is so closely linked to the underlying mechanisms [48,49], this might be a reason why it is so highly repeatable. Also in previous analyses with a larger sample size [36], we have shown repeatabilities of adult zebra finches to be 0.44 for TI latency after 48 ± 17 days (CI 0.21-0.56, p < 0.001, N = 144).

NO was not repeatable at all, probably due to different amounts of excitement induced by the types of objects. Birds reacted particularly strong with avoidance to the blue coloured objects used in rounds 4 and 6. About half of the birds did not approach the blue objects at all, but the ones that did reacted even more boldly, approaching it quicker or more often than the light coloured objects. This notion is also supported by the subadult stage, when only wooden and white coloured objects were used, where we found the highest repeatability estimate for NO among the three life stages (R = 0.28, but CI still overlapping with zero). We should have used objects that elicit similar states of excitement and did not consider beforehand that a bright blue object would be too different from the other ones. Yet our aim in using different objects was to avoid habituation which might have occurred after using the same object or too similar types of objects repeatedly.

### The time interval between tests influences repeatability

Longer intervals between tests are commonly expected to lead to decreased repeatabilities, for several reasons: firstly, because of environmental effects, as the animal is more likely to be in a similar state after a short interval. Secondly, the same is true for genetic influences (expression levels or epigenetic effects) on the phenotypic trait under study: after a longer interval the genetic changes might have been more pronounced. Thirdly, if the interval between tests even covers a major developmental re-organization such as sexual maturation (or a niche shift after metamorphosis e.g. [31]), it will probably also influence repeatability.

For instance, a study on male bush crickets (*Sciarasaga quadrata*) which were tested multiple times over their lifespan revealed significant repeatability in three parameters of calling activity throughout their lifespan, but a comparison of short-term and long-term intervals showed repeatability estimates decreasing over time [50].

Our study suggests differing results depending on the trait in question: in some cases, traits showed similar levels of short- and long-term repeatability (TI; AG in females) or were not repeatable at all (NO). In other cases, we found that traits were repeatable in the short-term (but also not in all life stages), but showed lower long-term repeatability (GA). Thus we found that repeatability estimates are not higher in general for short-term than long-term intervals, but it also has to be considered that our findings are based on a rather small sample size. The importance of taking multiple samples as suggested by Biro [51] becomes evident when comparing the repeatability estimates obtained for all six test rounds ('across lifestages') opposed to only rounds 1 and 6 ('long interval'). The power to detect significant repeatabilities if individuals are sampled over one year with only two measurements per individual seems to be too low in most cases (except TI).

### Development of repeatability across life stages

With a test-retest interval representing about 15-20% of zebra finches' life span, we were able to show that TI and NE were repeatable throughout.

In AG and GA, the repeatability estimates across life stages (rounds 1 to 6) were significant, although within life stages this was not always the case. This might be due to the bigger sample size across life stages compared with the within life stage subsets, allowing for more precise estimation of repeatabilities. This point is also supported by the generally lower repeatability estimates for the 'long interval' using only test rounds 1 and 6. The resulting large confidence intervals overlapping with zero may be an artefact, because with a lower number of measurements per individual the confidence interval tends to be overestimated [9]. In a previous study [36], we found position diversity index, one behavioural variable of GA, to be repeatable (R = 0.44, CI 0.11 - 0.64, p = 0.013, N = 77) in adult zebra finches tested with an interval of 148 ± 67 days.

The notion that personality traits undergo a gradual development at early life stages and become stable only thereafter has been challenged recently [29] and indeed we found no evidence for this to be the case. Our results rather suggest the opposite, as we did not find increasing or stabilizing repeatability estimates, but rather that traits were either in the same range of repeatability estimates in all life stages, or as in GA, birds were only showing repeatable behaviour at the earliest life stage but not anymore later on.

Other studies also found differences in repeatability across life stages and depending on the trait. For instance in yellow-bellied marmots (*Marmota flaviventris*), boldness was repeatable in only one age class (yearlings) and docility was repeatable in all three age classes (juveniles, yearlings, adults) [17].

One argument which contradicts the expectation of life-long stability is that proximate mechanisms, such as brain structure development, finishing only in adolescence, lead to rather gradual adjustments taking place during sensitive periods throughout ontogeny [29].

### Repeatability can differ between the sexes

Bell and co-workers [8] expected males to be more repeatable than females, which was confirmed in their overall meta-analysis. However, after exclusion of mate preference behaviours, females were the more repeatable sex.

We found that the sexes differed in repeatabilities, with males showing less repeatable behaviour than females. This resulted in overall repeatabilities being mainly driven by females' high estimates in the combined dataset, for instance in exploratory behaviour (NE). Females also became less repeatable in NE, while males became more repeatable (at the mature adult stage). Showing consistent exploratory behaviour might become important for zebra finch males at the adult stage after pair formation, as they are generally leading their females in feeding flocks [43].

In a study on domesticated female zebra finches [39], exploratory behaviour was repeatable over short-term (3 d and 7 d) and long-term (7 months) intervals. In contrast, another study [52] showed exploration was not repeatable in female zebra finches but in males. This suggests that either these differences result from the studies being undertaken with animals at different life stages or because there might be population differences in repeatability [53].

There is varying evidence regarding repeatability differences between the sexes in the literature. Supporting our finding that there was a tendency for lower repeatabilities in males, Burtka and Grindstaff [54] studied nest defence behaviour in Eastern bluebirds (*Siala sialis*) and found that females were more repeatable between years (within years also for one year, but not for the other). A study on field crickets (*Gryllus integer*) [55] reports that females were consistent in boldness (measured as latency to emerge from a refuge) tested across metamorphosis, while this was not the case for males. In common voles (*Microtus arvalis*), there were no sex differences in repeatabilities of boldness, exploration or activity [25].

The expectation to find sex differences in repeatability probably depends on the type of behaviour in general, but also on the species and their biology. Especially if traits are under sexual selection and related to mate choice, it might be advantageous to be predictable [56]. For instance, Nakagawa et al. [57] found that male house sparrows (*Passer domesticus*) were more repeatable than females (within and between years) in parental care behaviour (feeding rate).

### Development of behavioural syndrome structure over ontogeny

Three of the investigated traits formed a behavioural syndrome, but only in young adults. Thus, we found that the behavioural syndrome was not stable across life stages but specific for a certain age. Furthermore, it consisted only of non-repeatable traits and was mostly driven by the female sex.

At the young adult stage, the zebra finches were about 100 d old which is the time they are sexually mature and able to find a partner and reproduce, given favourable environmental conditions. This suggests that during the time of sexual maturation, traits are being restructured, so that previously independent traits become shortly linked together in young females when they reach reproductive age, but correlations then disappear again in mature animals. Young females that were more active also interacted more aggressively with the mirror, but were less bold towards a novel object. Possibly during the time when females are usually choosing a partner they become more active and more aggressive against female competitors. As the traits stayed uncoupled in males, there might be different selection regimes on the sexes.

It is expected that selection pressures on juveniles and adults differ significantly [27], leading to differences in personality axes as well, which in turn may influence the evolution of personality (e.g. if selection mainly acts on the juvenile stage). Differences in syndrome structure over ontogeny have been shown to occur in a number of study species. Bell and Stamps [15], in their study on two stickleback populations, found a stable boldness-aggression syndrome in one population, while the single behaviours constituting the syndrome were not repeatable. They also found (in the same population) correlations that were present during the juvenile stage, then disappeared in subadults but re-emerged in adults. In cavies, boldness and exploration were correlated in mature animals, but not in juveniles [24]. In wild brown trout, a behavioural syndrome including activity, aggression and exploration developed after an interval of two months covering a survival bottleneck [19]. In contrast, a stable syndrome structure comprising activity, exploration and boldness was detected in firebugs measured across life stage transitions [23]. In a meta-analysis on behavioural syndromes [20] the mean effect size was rather small (r = 0.264; 95% CI 0.210-0.316).

Thus it seems that behavioural syndromes are long-term consistent only in few cases, but can also quite readily change in others, with the correlations being broken or new ones forming over development. Changes in correlation structure can be expected to occur during development for several reasons, for instance because during major life stage transitions the most advantageous suite of behaviours changes. This is especially the case if the environment changes between age classes or if there is a prominent niche shift, as in the most extreme case of metamorphosis [1]. Another possibility is that when traits are affected by the same hormones, a shift in hormonal levels or a general hormonal reorganization such as during sexual maturation may influence the links between behavioural traits in such a way that they influence syndrome structure leading to instability [15]. On the other hand, it has been proposed that behavioural syndromes will be particularly stable if they are caused by genetic correlations, such as pleiotropy, or correlational selection [16,33]. However, the mechanisms generating, maintaining or disrupting behavioural syndromes are far from well understood and deserve further investigations.

We conclude that the stability of behavioural traits as well as their correlations cannot be assumed but need to be tested. This leads to the prediction that if single traits are regulated (more or less) independently from one another, animal personality might be much more flexible than expected so far, which is also supported by endocrinological findings [29]. Of course we are the first to admit that care needs to be taken as the low sample size, especially per sex, is a limitation in our study, meaning that we possibly could not detect further correlations due to this restriction [20]. Nevertheless we believe that more attention is needed when animal personality and behavioural syndromes are simply assumed to exist, rather than investigated.

## Conclusions

We could identify two traits that were very repeatable in zebra finches within and across life stages and in both sexes. TI and NE probably also represent independent personality axes, because they were never correlated with any of the other traits. However, we found no stable behavioural syndrome in our subset of wild-type zebra finches, but one has to emphasize that our sample size was rather low.

We stress the need for measurements on repeatability over larger time intervals and across ontogeny and agree with Stamps and Groothuis [27] that "[...] a 'snapshot' view of personality, which is based on descriptions of behaviour at a single age or life stage, provides an inadequate foundation for studies of personality across ecological and evolutionary scales of time and space." Further research should focus on multiple assessments of personality traits on a large time scale (relative to the life span of the study species), if possible over the course of development. This should be done ideally in a number of different personality traits, which allows estimation not only of the consistency of single personality traits, but also their functional coupling and if or how this changes over time. With our results, we wish to raise awareness that (animal) personality may not always be stable over the lifespan and that consistency of traits or syndrome structures might be overestimated.

Furthermore, one should be aware that repeatability is a population-level measure, and says nothing about within-individual change. It has been discussed that individual consistency may be a trait in itself [58,59] which has to be further investigated.

## Methods

### Animals and housing conditions

The zebra finch (*Taeniopygia guttata castanotis*) is a well-studied model organism [60] which can be bred and kept in captivity readily and has a short generation time, as juveniles are independent after 35 days and sexually mature after 65 days.

We conducted experiments on animal personality in zebra finches in a project on divergent bi-directional selection lines. The monitoring of the birds included five different personality tests, three of which served as selection parameters for the differing selection lines. The long-term design of the selection line study allowed us to assess repeatability and the development of behavioural syndromes over a longer time scale than is possible in most studies on long-lived organisms.

We chose 52 wild-type zebra finches from the F1-generation, 22 males and 30 females, to study long-term consistency of personality traits. They were bred from 31 different pairs, with between 5 and 14 individuals coming from the same selection line and maximum 3 full-sibs per family. They showed no behavioural trait values at the extreme end of the personality axis in the direction their parents were selected on.

The zebra finches were reared in outdoor aviaries (6 × 2 × 3 m) at Bielefeld University in the different selection line groups, each consisting of five breeding pairs plus their offspring.

After independence from their parents at ca. 40 days of age (median 43 d, min 30 d, max 62 d, IQR 8 d), subadult birds were translocated from their natal aviary groups into indoor mixed-sex tutor groups (cages: 81 × 60 × 50 cm). Tutor groups consisted of seven to ten juveniles with a pair of unrelated, unfamiliar adults. Before sexual maturation, at about 60 days of age (median 60 d, min 47 d, max 81 d, IQR 9 d), birds were then transferred to double cages (82 × 40 × 30 cm) in groups of three to four same-sex individuals. Tutor groups and same-sex cage groups were both arranged so they comprised animals originating from several different selection lines.

All animals were maintained in the same room with auditory and visual contact between cages. At all times, birds had *ad libitum *access to commercial zebra finch seed mix (Elles, Mischfutter für Exoten, L. Stroetmann Saat, 48163 Münster, Germany) and fresh water. Additionally, a mixture of germinated seeds and egg food (Cédé N.V., 9940 Evergem, Belgium) was provided daily in outdoor aviaries and tutor groups and thrice weekly after the transfer to double cages. The diet was weekly supplemented with fresh greens. In the housing room, birds were kept on a 14:10 light-dark-cycle, additional to the natural light conditions.

### Test schedule and description of behavioural tests

Behavioural tests started while birds were still kept in the tutor groups. All individuals were tested in five behavioural tests

(a) twice at the 'subadult' stage

(b) twice shortly after reaching adulthood ('young adult')

(c) twice at approximately one year of age ('mature adult')

leading to a total of six test rounds, with approximately 14 days apart within a given life stage. The interval between life stages was about one month for the transition to adulthood, and seven to ten months for the transition between young adult and mature adult. For all rounds, mean age and intervals between tests are given in table 1.

Experiments were conducted always in the same order: (TI - AG - GA - NO - NE) between 8:30 and 20:00 hours, and we took care that between tests, birds had at least one day break.

**Tonic immobility (TI): **An empty wire cage (40 × 31.5 × 21 cm) located on a table in a sound proof chamber (to exclude noise disturbances during the test) was used to conduct the tonic immobility test. The bird was placed on its back with wings pressed to its body on a metal holding cradle padded with foam rubber. An experimenter locked the bird in this position for five seconds by gently pressing index and middle fingers on its chest and then retracting the hands. Movements by the observer were minimized to prevent disturbances. Birds were considered successfully immobilized if they stayed in this position for at least five seconds. If a bird was not immobilized at once the procedure was repeated up to a maximum of 10 inductions. The test was terminated after a maximum of 20 minutes immobility (ceiling value of latency = 1200 s). Tests were performed by YW and student assistants Simon Tiersch and Nele Heitland (rounds 1-4) and Ivonne Kienast (rounds 5 and 6). Tonic immobility latencies and number of inductions are considered a measure of fearfulness [61] or boldness in predator contexts [62].

**Aggression (AG): **The aggression test cage (after [42]) was a small wire cage (40 × 31.5 × 21 cm) with one perch (39 cm). At the far end of the perch, a mirror (15 × 15 cm) was fixed and a food dispenser placed underneath.

The focal individual was transferred to the test cage with the mirror being covered with a piece of cardboard. After an habituation phase of five minutes, the mirror was uncovered and the recording started for five minutes. Birds were tested with the mirror fixed on either the right (= R) or left (= L) side of the cage in a given sequence (R-L-L-R-R-L). During the test, another cage of birds was present in the test room for auditory contact (always the same birds).

The first five seconds after removal of the division were discarded. Then, the frequency of the following interactions was determined within five minutes of video recording:

- Pecking/beak contact: each contact of the bill with the mirror.

- Flying against the mirror: body contact with the mirror while feet are not touching the perch.

- Breast contact: touching mirror with breast, usually after straightening up to full body height.

- Head contact: Usually occurring when sitting quite close to the mirror, it is touched with the head while the feet still remain on the perch.

We used the sum of aggressive interactions with the mirror as measure for aggressive behaviour.

**General activity (GA)**: The general activity test was conducted in the birds' home cage. All birds were removed from the cage prior to testing and relocated to a waiting cage (without visual contact to the focal individual). The food dispenser remained in the home cage during testing. After replacing the focal individual to its home cage and a two minute recovery time, the activities of the bird were video-recorded for ten minutes.

In the video analysis, the first 20 s were discarded (settling period). Then, the number of flights in the cage was determined. A flight was defined as a movement between perches or between perch and cage floor. We also determined the events of visits to all seven possible positions in the cage (four perches, left, middle and right bottom part of the cage) and calculated a position diversity index (PDI, equivalent to the calculation of the Exploration Diversity Index (EDI) as described in [53]). A higher position diversity index means that an animal visited more different places in the cage and thus indicates a higher activity.

**Novel object (NO): **Directly following the activity test, the divider was placed in the cage and a novel object inserted in the empty cage compartment. The position of the novel object (far left or far right perch) was randomly assigned beforehand. The novel object was fixed to a perch and placed inside the cage, while the focal individual was in the other compartment. The divider was then removed after the recording started, and the experimenter left the room. Behaviour was video-recorded for ten minutes.

This procedure was repeated with the remaining cage mates, while the already tested birds were transferred to the waiting cage.

As novel objects we used wooden blocks, styrofoam balls and chipboard discs of different colours. Details regarding the type of novel object used in each test round are given in part 6 in additional file 1.

Data of the activity test as well as the novel object data were analysed using CowLog [63]. The events and durations of visits to each of the seven positions in the cage were determined, as well as the latency to land on the novel object perch. We used the novel object latency, the frequency to visit the NO perch and the percentage of time spent there as variables for boldness. GA and NO tests were both conducted in the animal housing room.

**Novel environment (NE): **As a novel environment, we used standard double cages whose interiors were covered with adhesive foil. Otherwise, they were equipped as the usual home cages (sand on floor, four wooden perches, food dish with seed mixture, water bowl). Descriptions of the cage interiors used as novel environments are given in part 6 in additional file 1. Up to four individuals were tested simultaneously (in separate cages); in general all cage mates at a time. The focal individuals were transferred from their home cage into separated deprivation cages (30 × 40 × 40 cm) without food or water about 3.5 h (207 ± 10 min) prior to testing. Each focal individual was then put into a start box (11 × 17 × 11 cm) which was attached on either the left or right side of the experimental cage. A cardboard division between cages ensured that there was no visual contact between focal individuals in the start boxes. In the cages a food dish was placed on the same side as the start box, a water tray in the other compartment. The division between start box and novel environment was lifted and the behaviour video-recorded for one hour. A cage with flock mates was present in the experimental room during testing for auditory contact with the focal individuals.

As explorative behaviour, we determined how many of the seven possible positions in the cage were visited within one hour and we measured the latency to enter the novel environment cage and the latency to visit all seven positions if applicable. If the start box was not left or not all positions were explored, birds received a maximum value of 3601 seconds.

### Statistical analysis

All statistical analyses were run in statistical software R [64] within the RStudio environment [65], using the packages lme4, rptR, lattice, psych and psychometric [66-70].

### Principal components analysis

For each round per test we ran a principal component analysis (PCA) calculated with correlation matrix to extract the first axis for each personality trait. PCA were performed using the maximum available dataset (max. N = 52, for sample sizes per test and round see part 2, additional file 1). The included behavioural variables and resulting PC loadings are given in part 2 in additional file 1. Because seven data points from six individuals were missing, the final dataset for subsequent analyses was reduced to 46 individuals (n = 276). For general activity (GA) round 1, the derived scores were inverted by multiplication with (-1), so that higher scores represent bolder behaviours in all tests and rounds (GA = more active; NO = bolder towards object; NE = faster exploration; TI = lower latency and more inductions; AG = more aggressive). As the loadings differed between test rounds for aggression (AG) and the derived PC scores were not normally distributed, we instead used the decimal logarithm of the sum of aggressive interactions with the mirror as a score for later analysis, which showed a distribution closer to normality.

### Generalized Linear Mixed Models for extraction of best linear unbiased predictors

For each personality axis in each life stage, we ran a univariate Generalized Linear Mixed Model (GLMM) with Gaussian error structure fit with Maximum Likelihood. This was done to extract the best linear unbiased predictors (BLUPs) for behavioural syndrome structure calculation and to check for effects of fixed factors such as sex differences in mean level behaviour.

Models were run in R using the function lmer from the R package lme4 [66]. In the full model we included the three fixed effects sex, test round and selection line and the two-way interaction of sex and test round as well as the random effects batch (to control for possible season effects, as all birds from the F1-generation were transferred from outdoor aviaries to indoor cages in a total of 10 batches between mid-June and mid-December 2012) and individual ID nested within mother ID. We conducted a stepwise deletion of fixed effects if model comparison using a likelihood-ratio analysis between the full and reduced model was non-significant. The random effects as well as test round and selection line as a fixed effect to control for were always retained in the final model.

Because Fligner-Kileen tests indicated heterogeneity of variance in NO scores for the interaction of the fixed factors sex and round, we weighted this effect (by the ratio between the variances of the factor levels) to improve the fit of the model. NO models were calculated with and without weights and the model results for both are presented in parts 4 and 5 in additional file 1. Although likelihood-ratio tests showed that the weights significantly improved the fit, we decided to use the unweighted models for subsequent analyses after visual inspection of the residuals plotted against the fitted values.

Because of ceiling or floor effects of the data distribution, we created subsets of data containing only valid data points (without zero- or maximum-inflated data) as follows: TI was maximum-inflated as individuals that did not enter the immobile state after ten inductions received a latency of one second (leading to high PC scores). The reduced dataset included data points with latencies larger than one. AG, NO, NE and GA all were zero-inflated, as some individuals did not interact with the mirror (interactions = 0), not approach the novel object (events on perch = 0), not explore all seven positions the novel environment (received a maximum latency of 3601 s resulting in lowest PC scores) or not move in the home cage (flights = 0) at all. In the respective reduced datasets these data points were removed. Each model (one per life stage and personality trait) was run for the inflated dataset and reduced dataset separately and the model outputs are given in parts 4 and 5 in additional file 1. Although results of the reduced dataset deviated from results of the inflated dataset in some cases, we continued calculations with the complete (inflated) dataset, as using the reduced dataset would have decreased our overall sample size (see part 5 additional file 1 for reduced dataset sample sizes) immensely. We discuss these cases carefully.

### Repeatabilities ('differential consistency')

Repeatability is calculated as the ratio of two variances, the variance within groups and the variance among groups as follows:

$$R=\frac{V_{among\;groups}}{V_{among\;groups}+V_{within\;groups}}$$

We used the R package rptR [67] to calculate repeatabilities within and between life stages. All response variables (PC axes and logarithmized sum of aggressive interactions) were approximately normally distributed and thus analysed with Gaussian fit and REML estimation. We report repeatability estimates with their significance levels and 95% confidence intervals, calculated with 1000 permutations and 1000 bootstrappings. We calculated repeatabilities within each life stage (comprising two subsequent test rounds), for the complete dataset including all six test rounds (referred to as 'across life stages') and also including only test rounds 1 and 6, to get an estimate across life stages using only two data points (referred to as 'long interval'). We compared the overlap of confidence intervals, showing non-significance at the 5% threshold if applicable [71]. As the mixed models had indicated a significant effect of sex in some cases, we calculated repeatabilities for males and females separately as well.

### Behavioural syndrome structure ('structural consistency')

We extracted the best linear unbiased predictors (BLUPs) of the GLMMs. BLUPs represent scores that are "controlling for" the effects included in the model. Behavioural syndrome structure at the three life stages was assessed by computing Spearman rank correlation matrices with Holm's correction using the life stage mean of the fitted values of the minimum adequate models of the five personality traits for each life stage (R package psych [69]). The lower and upper bounds of 95% confidence intervals were estimated with the R package psychometric [70]. We also calculated behavioural syndrome structure separately for males and females.

## Ethics statement

Housing of birds was permitted by the veterinary office Bielefeld, Germany (# 530.421630-1, 18.04.2002 and # 530.4, 27.07.2014) and experiments were conducted according to the German law for experimentation with animals.

## Declarations

This research was funded by a grant from the German Research Foundation to OK (DFG, KR 2089/2-1). Publication costs for this article were funded by the German Research Foundation (FOR 1232) and the Open Access Publication Fund of Bielefeld and Muenster University.

## Competing interests

The authors declare that they have no competing interests.

## Authors' contributions

YW and OK conceived and designed experiments. YW performed the experiments and analyzed the data. YW and OK wrote the paper. All authors read and approved the final manuscript.

## Supplementary Material

Additional file 1 Supplementary Material Click here for file
