# Supplementary material for: Personality over ontogeny in zebra finches: long-term repeatable traits but unstable behavioural syndromes
Source: Front Zool. 2015 Aug 24;12(Suppl 1):S9. doi: 10.1186/1742-9994-12-S1-S9 (PMC4722341; doi:10.1186/1742-9994-12-S1-S9)
Supplement: Additional file 1 — Supplementary Material [file 1742-9994-12-S1-S9-S1.docx]

**Supplementary Material**

**Part 1 – Descriptive statistics for all variables of the five tests (mean ± SD).** The raw, untransformed variable means are given separately for males and females for each of the six test rounds. All variables are counts, except for PDI (ratio) and Percentage NO perch (percentage).

|  |  |  | subadult | | young adult | | mature adult | |
| --- | --- | --- | --- | --- | --- | --- | --- | --- |
| Test | Variable (untransformed) | sex | Round 1 | Round 2 | Round 3 | Round 4 | Round 5 | Round 6 |
| Tonic Immobility (TI) | Latency [s] | female | 81 ± 85 | 64 ± 74 | 47 ± 40 | 63 ± 53 | 65 ± 79 | 118 ± 222 |
|  |  | male | 153 ± 169 | 93 ± 131 | 73 ± 87 | 65 ± 111 | 78 ± 86 | 77 ± 105 |
|  | Trials | female | 3 ± 2 | 3 ± 3 | 3 ± 3 | 2 ± 2 | 3 ± 3 | 4 ± 3 |
|  |  | male | 3 ± 3 | 3 ± 3 | 3 ± 3 | 4 ± 3 | 3 ± 3 | 5 ± 4 |
| Aggression (AG) | Pecks | female | 12 ± 23 | 12 ± 21 | 29 ± 68 | 14 ± 25 | 9 ± 23 | 17 ± 46 |
|  |  | male | 12 ± 18 | 9 ± 20 | 21 ± 41 | 8 ± 10 | 11 ± 18 | 7 ± 14 |
|  | Flights | female | 3 ± 5 | 2 ± 3 | 1 ± 2 | 1 ± 3 | 1 ± 1 | 0 ± 1 |
|  |  | male | 2 ± 4 | 3 ± 8 | 2 ± 4 | 1 ± 3 | 11 ± 38 | 1 ± 2 |
|  | Head contacts | female | 0.04 ± 0.4 | 0.04 ± 0.2 | 0 ± 1 | 0 ± 1 | 0 ± 0 | 0 ± 1 |
|  |  | male | 0 ± 1 | 1 ± 2 | 0 ± 1 | 0.1 ± 0.4 | 0 ± 1 | 0 ± 1 |
|  | Breast contacts | female | 0.08 ± 0.4 | 1 ± 2 | 0 ± 1 | 0 ± 1 | 0 ± 1 | 0 ± 1 |
|  |  | male | 1 ± 2 | 0.1 ± 0.5 | 1 ± 2 | 0.1 ± 0.3 | 0 ± 1 | 4 ± 16 |
|  | Sum of interactions | female | 14 ± 27 | 14 ± 24 | 31 ± 70 | 16 ± 26 | 10 ± 26 | 18 ± 47 |
|  |  | male | 14 ± 21 | 13 ± 29 | 24 ± 44 | 9 ± 11 | 22 ± 43 | 11 ± 22 |
| General Activity (GA) | Flights | female | 45 ± 45 | 63 ±97 | 41 ± 60 | 18 ± 28 | 38 ± 52 | 46 ± 58 |
|  |  | male | 52 ± 54 | 75 ± 103 | 68 ± 70 | 40 ± 42 | 31 ± 40 | 37 ± 55 |
|  | Position Diversity Index (PDI) | female | 0.98 ± 0.54 | 0.90 ± 0.57 | 0.92 ± 0.58 | 0.62 ± 0.57 | 1.05 ± 0.54 | 1.03 ± 0.54 |
|  |  | male | 0.98 ± 0.59 | 1.01 ± 0.51 | 1.06 ± 0.48 | 0.94 ± 0.52 | 1.21 ± 0.39 | 0.99 ± 0.51 |
| Novel Object (NO) | Latency object [s] | female | 182 ± 129 | 63 ± 109 | 104 ± 122 | 254 ± 100 | 99 ± 133 | 242 ± 107 |
|  |  | male | 160 ± 139 | 45 ± 94 | 90 ± 120 | 253 ± 99 | 90 ± 137 | 238 ± 116 |
|  | Events NO perch | female | 2 ± 4 | 12 ± 17 | 10 ± 12 | 1 ± 2 | 4 ± 7 | 1 ± 3 |
|  |  | male | 5 ± 7 | 21 ± 27 | 18 ± 19 | 1 ± 3 | 8 ± 13 | 1 ± 4 |
|  | Percentage NO perch | female | 1.35 ± 3.11 | 18.33 ± 28.74 | 10.05 ± 12.23 | 5.78 ± 20.71 | 2.47 ± 6.27 | 0.56 ± 1.52 |
|  |  | male | 5.92 ± 14.62 | 18.21 ± 21.49 | 13.59 ± 20.37 | 0.25 ± 0.91 | 8.38 ± 20.73 | 0.64 ± 1.70 |
| Novel Environment (NE) | Number of positions visited | female | 6 ± 1 | 6 ± 2 | 6 ± 2 | 6 ± 1 | 6 ± 1 | 6 ± 1 |
|  |  | male | 7 ± 1 | 7 ± 0.4 | 7 ± 1 | 7 ± 0.5 | 7 ± 0.6 | 7 ± 1 |
|  | Latency all positions [s] | female | 2493 ± 1000 | 1922 ± 1380 | 1745 ± 1549 | 1955 ± 1627 | 2128 ± 1444 | 2843 ± 1097 |
|  |  | male | 2100 ± 1149 | 1393 ± 1327 | 1172 ± 1360 | 2136 ± 1520 | 1637 ± 1533 | 1550 ± 1535 |
|  | Latency Box [s] | female | 12 ± 57 | 1 ± 2 | 1 ± 1 | 1 ± 1 | 1 ± 2 | 2 ± 5 |
|  |  | male | 3 ± 5 | 1 ± 1 | 1 ± 1 | 1 ± 1 | 2 ± 3 | 1 ± 2 |

### Part 2 – Variables and loadings of principal components analyses of the five tests. Variables marked with ^4^ were 4th root transformed before PCA. Scores of GA in test round 1 were multiplied with (-1) so that the sign of PC scores in all six rounds are equivalent. In all tests except for AG, higher PC scores represent bolder behaviours

|  |  | Component loadings | | | | | |
| --- | --- | --- | --- | --- | --- | --- | --- |
|  |  | subadult | | young adult | | mature adult | |
| Test | Variables | Round 1 | Round 2 | Round 3 | Round 4 | Round 5 | Round 6 |
| Fearlessness (TI) | Latency^4^ | -0.707 | -0.707 | -0.707 | -0.707 | -0.707 | -0.707 |
|  | Trials | 0.707 | 0.707 | 0.707 | 0.707 | 0.707 | 0.707 |
|  | cumulative variance explained [%] | 73.0 | 73.5 | 74.0 | 73.0 | 83.5 | 77.9 |
|  | N = | 50 | 51 | 52 | 51 | 52 | 52 |
| Aggression (AG) | Flights | -0.614 | -0.553 | 0.587 | -0.010 | -0.148 | -0.223 |
|  | Pecks | -0.692 | -0.563 | 0.522 | -0.587 | -0.678 | -0.717 |
|  | Head contacts | -0.268 | -0.529 | 0.532 | -0.568 | -0.486 | -0.656 |
|  | Breast contacts | -0.268 | -0.311 | 0.315 | -0.577 | -0.531 | 0.072 |
|  | cumulative variance explained [%] | 40.7 | 57.3 | 43.9 | 55.9 | 44.6 | 34.6 |
|  | N = | 52 | 52 | 52 | 52 | 52 | 52 |
| Activity (GA) | Flights^4^ | -0.707 | 0.707 | 0.707 | 0.707 | 0.707 | 0.707 |
|  | Position Diversity Index (PDI) | -0.707 | 0.707 | 0.707 | 0.707 | 0.707 | 0.707 |
|  | cumulative variance explained [%] | 94.2 | 92.3 | 93.2 | 93.3 | 87.4 | 86.9 |
|  | N = | 52 | 52 | 52 | 52 | 52 | 51 |
| Boldness (NO) | Latency object | 0.566 | 0.557 | 0.573 | 0.564 | 0.568 | 0.571 |
|  | Events NO perch^4^ | -0.592 | -0.590 | -0.592 | -0.597 | -0.593 | -0.582 |
|  | Percentage NO perch^4^ | -0.573 | -0.585 | -0.566 | -0.570 | -0.571 | -0.579 |
|  | cumulative variance explained [%] | 92.3 | 82.9 | 82.2 | 86.5 | 89.1 | 97.0 |
|  | N = | 52 | 52 | 52 | 52 | 52 | 50 |
| Exploration (NE) | Number of positions visited | 0.707 | 0.655 | 0.614 | 0.649 | 0.673 | 0.705 |
|  | Latency all positions | -0.696 | -0.617 | -0.679 | -0.694 | -0.664 | -0.701 |
|  | Latency Box | 0.122 | -0.436 | -0.402 | -0.313 | -0.324 | 0.102 |
|  | cumulative variance explained [%] | 52.6 | 60.0 | 56.3 | 56.0 | 59.8 | 55.8 |
|  | N = | 52 | 52 | 52 | 52 | 52 | 52 |

**Part 3 – Behavioural syndrome structure at the three life stages, calculated separately for males (above diagonal, N = 21) and females (below diagonal, N = 25).** Spearman rank correlations calculated for each life stage with the mean of fitted values from the models (BLUPs). P-values were adjusted for multiple testing with Holm’s correction. Significant correlations are in bold. *p < 0.05; ** p < 0.01

| Subadult | | | | | |
| --- | --- | --- | --- | --- | --- |
|  | TI | AG | GA | NO | NE |
| Fearlessness (TI) |  | 0.04 | 0.14 | 0.20 | -0.09 |
| Aggression (AG) | 0.12 |  | 0.04 | 0.18 | -0.04 |
| General activity (GA) | 0.04 | 0.10 |  | 0.19 | 0.00 |
| Boldness (NO) | -0.13 | 0.13 | 0.48 |  | 0.38 |
| Exploration (NE) | 0.10 | -0.06 | 0.23 | 0.08 |  |
| Young adult | | | | | |
|  | TI | AG | GA | NO | NE |
| Fearlessness (TI) |  | -0.05 | -0.47 | 0.28 | 0.21 |
| Aggression (AG) | 0.01 |  | 0.44 | -0.38 | 0.19 |
| General activity (GA) | -0.32 | **0.53*** |  | -0.54 | -0.08 |
| Boldness (NO) | 0.16 | **-0.69**** | **-0.61**** |  | 0.35 |
| Exploration (NE) | -0.10 | -0.05 | -0.13 | 0.31 |  |
| Mature adult | | | | | |
|  | TI | AG | GA | NO | NE |
| Fearlessness (TI) |  | 0.06 | 0.15 | 0.09 | -0.11 |
| Aggression (AG) | -0.21 |  | 0.00 | 0.14 | 0.57 |
| General activity (GA) | -0.35 | -0.33 |  | 0.11 | 0.34 |
| Boldness (NO) | -0.03 | 0.26 | 0.13 |  | 0.24 |
| Exploration (NE) | 0.04 | -0.01 | 0.03 | -0.05 |  |


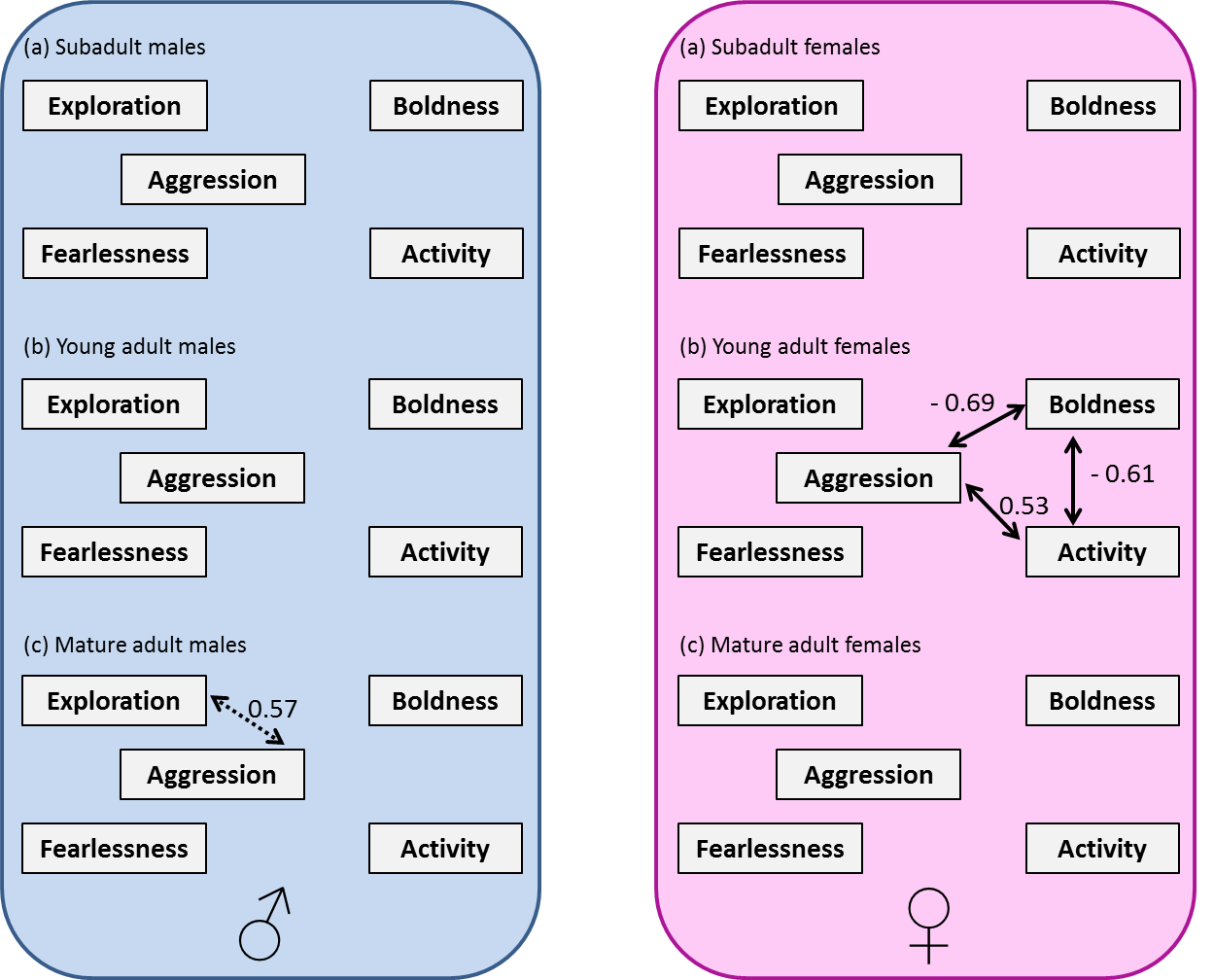


**Part 4 – Generalized linear mixed effects models for the five personality traits at the subadult, young adult and mature adult life stages calculated for the inflated dataset (n = 92 observations, n groups: ID nested in mother = 46, mother = 27, batch = 9)**

**part 5 – Generalized linear mixed effects models for the five personality traits at the subadult, young adult and mature adult life stages, calculated with reduced datasets (of different sample sizes) after removal of ceiling or floor values**

**part 6 – Description of novel objects and environments used in the different test rounds**

| Life stage | Round | Details of novel objects | Details of novel environments |
| --- | --- | --- | --- |
| Subadult | 1 | wooden block made of pine  (3 x 5 x 3 cm) | black and white zebra pattern  (d-c-fix self-adhesive foil, Konrad Hornschuch AG) |
|  | 2 | wooden block, painted white  (3 x 5 x 3 cm) | black and white chessboard pattern (d-c-fix self-adhesive foil, Konrad Hornschuch AG) |
| Young adult | 3 | white styrofoam ball  (diameter 4 cm) | chessboard pattern plus two pieces of cardboard attached to cage walls (pink triangle 14.8 x 12.8 x 12.8 cm and yellow square 21 x 21 cm) |
|  | 4 | styrofoam ball painted blue (diameter 4 cm) | zebra pattern plus two pieces of cardboard attached to cage walls (blue circle with diameter 9 cm and yellow square 15 x 15 cm) |
| Mature adult | 5 | chipboard disc painted white (diameter 7.5 cm, 5 mm thick) | horizontal stripe pattern made of black tape stripes (3 cm width) |
|  | 6 | chipboard disc painted blue (diameter 7.5 cm, 5 mm thick) | pattern of black half circles (diameter 8 cm) attached to cage walls |
